# Interrater reliability of physical examination tests in the acute phase of shoulder injuries

**DOI:** 10.1186/s12891-021-04659-x

**Published:** 2021-09-09

**Authors:** Malte Schmidt, Martine Enger, Are Hugo Pripp, Lars Nordsletten, Stefan Moosmayer, Knut Melhuus, Jens Ivar Brox

**Affiliations:** 1 Department of Orthopaedic Emergency, Division of Orthopaedic Surgery, Oslo University Hospital, Postboks 4956 Nydalen, 0424 Oslo, Norway; 2 Institute of Clinical Medicine, University of Oslo, Oslo, Norway; 3 Oslo Centre of Biostatistics and Epidemiology, Oslo University Hospital, Oslo, Norway; 4 Department of Orthopaedic Surgery, Martina Hansens Hospital, Sandvika, Norway; 5 Department of Physical Medicine and Rehabilitation, Oslo University Hospital, Oslo, Norway

**Keywords:** Reliability, Agreement, Physical examination test, Acute shoulder injury

## Abstract

**Background:**

The physical examination is one of the cornerstones of the diagnostic process in patients with acute shoulder injuries. The discriminative properties of a given examination test depend both on its validity and reliability. The aim of the present study was to assess the interrater reliability of 13 physical examination manoeuvres for acute rotator cuff tears in patients with acute soft tissue shoulder injuries.

**Methods:**

In a large walk-in orthopaedic emergency department, 120 consecutive patients ≥40 years of age were included in a diagnostic study. Patients who had follow-up within three weeks of an acute shoulder injury without fracture on radiographs were eligible. Four emergency department physicians participated as examiners. In a subset of 48 patients, the physical examination tests were performed by two physicians, randomly chosen by their work rotation. The physicians were blinded to the findings of each other and the results of the ultrasound screening. The interrater reliability was assessed by Cohen’s kappa, intraclass correlation coefficient (ICC), standard error of measurement (SEM) and Bland-Altman plots depending on whether the examination test result was registered as a binary, ordered categorical or continuous numerical variable.

**Results:**

The median age was 55.5 years, 46% were female. Twenty-seven percent had a rotator cuff full-thickness tear on ultrasound screening; all but one involved the supraspinatus tendon. Cohen’s kappa for binary tests ranged from excellent to fair. Excellent agreement (kappa > 0.8) was found for the inability to abduct above 90° and abduction strength. External rotation strength expressed substantial agreement (kappa 0.7). The lowest scores were registered for Hawkins` test and the external rotation lag sign (kappa 0.25 and 0.40, respectively). The ICCs for active range of abduction and external rotation were 0.93 (0.88–0.96) and 0.84 (0.72–0.91), whereas the SEM was 15 and 9, respectively.

**Conclusions:**

The results indicate that examination manoeuvres assessing abduction and external rotation range of motion and strength are more reliable than manoeuvres assessing pain in patients in the acute phase of traumatic shoulder injury. The poor agreement observed is likely to limit the validity in the present setting of two commonly used tests.

**Trial registration:**

The Norwegian Regional Ethics Committee South East (2015/195).

**Supplementary Information:**

The online version contains supplementary material available at 10.1186/s12891-021-04659-x.

## Background

A careful history and a systematic clinical examination are cornerstones for the evaluation of patients with shoulder pain [1]. The diagnostic value of the clinical examination depends upon the skills of the examiner and the reliability and validity of the clinical tests. Previous studies and reviews have to a large degree focused on the validity of physical examination tests, and reviews have concluded that there is insufficient evidence upon which to make clinical recommendations [2–6].

One possible reason for the limited diagnostic accuracy observed, would be that the intra- and interrater reliability and agreement of tests were low. There is however a paucity of high quality studies addressing this issue [5, 7, 8]. Furthermore, Lange’s review and meta-analysis in 2016 pointed to the heterogeneity of reliability measurements hindering proper synthesis of the data [8]. Interrater reliability of the Cyriax based clinical tests has previously been reported to be good to excellent [9, 10], but a recent evaluation of these tests in general practice found poor to moderate interrater agreement [11]. This discrepancy may depend on the selection of tests and examiners, as well as the methodology of the studies.

The accuracy of clinical shoulder tests in diagnosing rotator cuff disorders has been investigated in numerous studies [5, 6, 12, 13]. However, a common feature of most of these studies is that experienced examiners, often with shoulder disorders as their specialty or field of interest, performed the tests. We wished to evaluate the tests when performed by physicians outside of the tertiary health care system, where most patients are.

The aim of the present study was to explore the interrater reliability of physical examination shoulder tests aiming to diagnose acute rotator cuff lesions in patients with previously healthy shoulders who had sustained an acute soft tissue shoulder injury.

## Patients and methods

### Patients

**Fig. 1:**
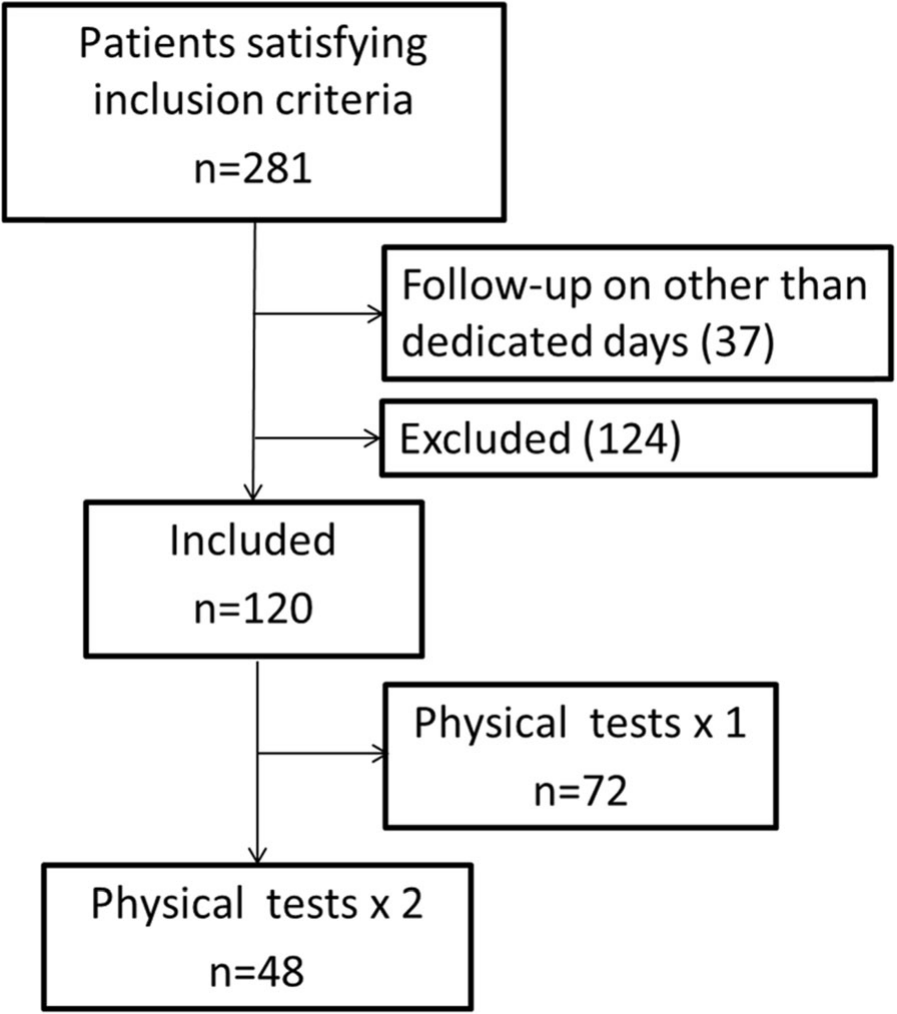
Flow of participants

The present study is a subset of a diagnostic accuracy study of 120 patients 40 years or older, who had follow-up at the Department of Orthopaedic Emergency, Oslo University Hospital within 3 weeks of an acute shoulder injury. The facility is a combined primary and secondary care emergency department admitting non-referred patients. The department’s treatment algorithm recommends follow-up for patients with at least one of the following: pain intensity of 4 or more at rest or during activity on a numeric rating scale from zero to ten (worst pain), abduction active range of motion reduced by > 30° or external rotation active range of motion reduced by > 20° (additional file 1). Inclusion criteria were acute soft tissue shoulder injury or successfully reduced glenohumeral dislocation with a concomitant onset of symptoms and no fracture on plain radiographs. Exclusion criteria were injury of both shoulders, previous shoulder surgery during last 6 months, known rotator cuff tear on imaging, ongoing neck−/shoulder problems and other serious disease. One hundred and twenty consecutive patients were included, of which 48 were examined by two physicians and included in the present study (Fig. 1). The 48 included patients were randomly selected by the department’s work rotation: during the inclusion period of the present study, a second examiner performed the tests in addition to the first if at least two of the four participating physicians were present at the facility.

Age, gender and injury mechanism were recorded. The patients filled in the Oxford Shoulder Score (OSS) ranging from 0 (most severe symptoms) to 48 (least symptoms) at inclusion.

The study was approved by the Norwegian Regional Ethics Committee South East (2015/195) and performed in accordance with the Helsinki declaration. Written informed consent was obtained from all participants. The study was registered in ClinicalTrials.gov with ID: NCT02644564.

### Clinical tests

Four physicians, none of whom were specializing in shoulder disorders, performed the clinical tests. They had from 1.5 to 6 years of experience at Department of Orthopaedic Emergency. The physicians were given 30 min instruction and written information on the testing procedures (additional file 2). They were blinded to the findings of each other and to the ultrasound screening which was the reference standard. The second author who performed the ultrasound screening, had undergone formal training and had performed 4–6 scans per week for 1.5 years when the study started. The ultrasound screening was performed according to a standard protocol [14, 15]. In 53 of the 120 patients of the cohort, MRI was performed. There was disagreement between the MRI and the ultrasound regarding the target condition full-thickness tear in 2 cases (4%).

The ultrasound and physical examination test results were recorded in structured questionnaires as well as in the patient records. The patients were independently examined by two of the four physicians at the first follow-up consultation when inclusion took place. They had clinical information available by the inclusion criteria and were also informed as to whether the patient had sustained a glenohumeral dislocation. The examiners did not read the electronic patient record notes from the primary visit, as previous examination results might influence the interpretation of the tests. The time interval between the two assessors was less than 1 h.

The target condition that the physical examination tests aimed to detect was acute rotator cuff full-thickness tears. Occult fractures of the tendon insertion were included in the target condition, as a physical examination test could not be expected to discriminate between an avulsion of the tendon insertion and a tear of the tendon itself [16]. An occult fracture was defined as a fracture that could not be identified on the primary plain radiographs by the physician in charge or by the skeletal radiologist [17]. The tests used were chosen because of the accuracy reported in articles, reviews and meta-analyses [6, 13, 18–23], the probability of patients being able to execute the tests in an acute setting, as well as the feasibility of the tests in emergency departments and general practice.

The tests performed in the scope of this study are presented in Table 1. Range of motion and strength were assessed clinically without the use of goniometers or dynamometers as they are not in common use in emergency departments and primary health care. In accordance with the department’s routine, abduction above 90° and maximal external rotation were not performed at first follow-up in patients with glenohumeral dislocation. These patients were not included in the reliability analysis of the relevant tests (inability to abduct > 90°, painful arc, external rotation active range of motion (AROM) reduction and lag sign).

**Table 1:** Physical examination tests assessed for acute rotator cuff full-thickness tear in soft tissue shoulder injuries

Test	Scope of test	Test method	Positive test	References	
				Test method	Test choice
Abduction AROM	Supraspinatus muscle or occult injury of greater tuberosity	The patient is asked to raise both arms to the side and up. Demonstrated by examiner in the scapular plane (20°- 30° in front of coronal plane), thumbs upwards to minimise pain.	Inability to abduct > 90° (derived from the registered no of degrees of abduction)	[12]	[12, 21]
Painful arc	Supraspinatus muscle or occult injury of greater tuberosity (“Impingement”)	As over, active test (patient raises the arm)	Pain between 60 ° - 120° abduction localised to the deltoid region	Kessel and Watson, 1977	[19, 20]
Abduction strength	Supraspinatus muscle or occult injury of greater tuberosity	Upper arm along side, elbow in 90° flexion, isometric test of abduction strength at 0° and 45° of abduction	Strength reduced compared to uninjured side		*
Resisted abduction pain	Supraspinatus muscle, occult injury of greater tuberosity	Like for abduction strength, but the arm is passively moved to 30°- 40° abduction. If there is no pain by holding the arm in this position, resistance to abduction is applied increasingly (isometric, eccentric).	Pain against gravity or isometric resistance. (Negative test: no pain or pain on eccentric resistance)		*
Hawkins` test	Supraspinatus muscle or occult injury of greater tuberosity (“Impingement”)	The arm is brought to approximately 90° forward flexion, with elbow flexed 90°. The scapula is stabilized with one hand, while the other stabilizes the elbow and internally rotates the shoulder.	Pain or marked worsening of existing pain on internal rotation	Hawkins, 1980	[19, 20]
External rotation AROM	Infraspinatus muscle or occult injury of greater tuberosity	The patient stands with back against wall, elbows flexed 90° and held along the side of the body. The patient actively rotates the arm externally while the examiner demonstrates.	≥ 20° difference between sides (derived from registered no of degrees)		@
External rotation strength	Infraspinatus muscle or occult injury of greater tuberosity	Starting position as over, strength of the patient’s external rotation evaluated with examiner’s hand resisting proximal to the patients wrist	Reduced strength compared with uninjured side	[12]	[12, 23]
Small finger test	Infraspinatus muscle or occult injury of greater tuberosity	Starting position as over. The examiner stands on the patient’s side and attempts to push with internal rotation force against patient’s wrist using only his or her small finger	Cannot resist examiner’s force		#
External rotation lag sign	Infraspinatus muscle	Elbow flexed 90°. Elbow supported by examiner’s hand and brought to slight elevation (about 20°) in the scapular plane. The examiner’s other hand externally rotates the arm to maximum position, then lets up slightly (about5°). The patient is asked to keep the position when the examiner lets go of the wrist, but still supports the elbow.	Unable to hold position	[12]	[12, 19]
Internal rotation AROM	Subscapularis muscle or occult injury of lesser tuberosity	Attempt to bring hand behind the body and as high as possible on the back	Different level (of four §) compared with uninjured side		
Belly-Press	Subscapularis muscle or occult injury of lesser tuberosity	Patient exerts pressure on the abdomen with flat hands and with the arm in maximum internal rotation (elbows in front of trunk and straight wrists). May be enforced by the examiner applying external rotation force	Cannot hold position or reduced strength compared with uninjured side	[22]	[22]
Internal rotation lag sign	Subscapularis muscle or occult injury of lesser tuberosity	Shoulder passively extended and internally rotated to maximum, with elbow flexed 90°	Unable to hold position	[22]	[6, 12, 19]
Internal rotation lag sign anteriorly	Subscapularis muscle or occult injury of lesser tuberosity	Hands on belly, elbows passively led in front of body until maximum internal rotation in shoulder	Unable to hold position		¤

*AROM* active range of motion; § 0-plane, gluteal area, lumbar area or inter-scapular area *Strength was assessed in this way, as a large proportion of the patients could not abduct to 90°, and hence normal strength tests like the Dropping sign, Supraspinatus/Empty can/Jobe’s test or the Full can test could not be performed. @ Goniometers are not normally in use in the first line services. This position reduces the risk of misinterpreting the number of degrees due to patient rotating the spine. ^#^ Test traditionally used in the hospital, not previously published. ¤ Test included due to some acutely injured patients being unable to perform the belly-press and internal rotation lag sign

### Statistics

A sample size of 48 was comparable with other relevant studies and found adequate [24–27]. To evaluate interrater reliability for dichotomous variables, Cohen’s kappa was used [28]. Kappa statistics expresses the degree of agreement between two raters corrected for chance agreement [29]. A value of − 1 represents absolute disagreement, a value of 0 no agreement above chance, and a value of 1 absolute agreement. There is no value of k that can be regarded as a universal indicator of good agreement, and individual interpretation is recommended. Previous studies have considered values ≤0.4 as fair to poor, from 0.41–0.60 as moderate, 0.61–0.80 substantial and values greater than 0.80 as excellent or almost perfect [30]. Linear weighted kappa was used for the ordered categorical variable internal rotation active range of motion that had four categories (Table 1).

To allow for a more diverse interpretation of agreement we also calculated the percentage of absolute agreement by dividing the number of cases in which both raters agreed with the total number of cases.

For continuous numerical variables (degrees of external rotation and abduction) the intraclass correlation coefficient (ICC (1,1); one-way random, single measures in SPSS) and standard error of measurement (SEM) were calculated. Under the conditions of the present study with a sample of more than 30 heterogeneous patients and more than 3 raters, ICC values from 0.5 to 0.75 suggest moderate reliability, 0.75 to 0.9 good, and above 0.9 excellent reliability [27]. For the SEM, the standard deviation (SD) of the measurements (subjects) were estimated by first calculating the mean of the SD of the first and second raters` results. The SEM was then calculated as the SD x √(1-ICC).

Bland-Altman plots were used to assess the mean difference and the limits of agreement between raters [31]. Heteroscedasticity was examined by visual inspection of the plots, whereas linear regression analysis was performed to control for proportional bias of the continuous variables.

We compared the demographic data of the subset examined by two physicians with the remainder in the main study using the Chi-square and Mann-Whitney-U-test.

IBM SPSS Statistics Version 23 was used for all analyses apart for SEM for which Version 26 was used.

## Results

A total of 48 patients were included in this analysis. The median age was 55.5 years (interquartile range (IQR) 46–64) and 46% were female. The age and sex distribution was not different from the other 72 patients of the main study. The mean number of days from the accident to inclusion and examination was 12 (SD, 3.4), and 85% were injured due to falls. The mean Oxford Shoulder Score was 27.5 (SD, 8.7) at inclusion.

The proportion of patients with a full-thickness rotator cuff tear was 27% (*n* = 13) and also not different from the main study. All but one tear involved the supraspinatus, and in five cases the tear extended into the superior portion of the subscapularis tendon. There was one isolated superior full-thickness subscapularis tendon tear, but no full-thickness, full-width tears. Furthermore, 8 patients (17%) had sustained a glenohumeral dislocation, whereas 25 were classified as contusions or sprains. The remaining patients had occult fractures (*n* = 4), sternoclavicular dislocation (*n* = 1) or a tear of the long head of the biceps (*n* = 1). Four patients had two diagnoses.

The valid number of comparisons is presented in Table 2. Six patients that had a recent shoulder dislocation were prohibited by department protocol to abduct > 90°. Those that abducted to 90° were therefore excluded from the analysis of the inability to abduct > 90° and the painful arc tests, as they could potentially be interpreted as false positives. The observed range of motion extended from 0 to 180 degrees for abduction, and from 0 to 90 degrees for external rotation.

**Table 2:** Inter-observer reliability of physical examination tests in patients diagnosed as acute soft tissue shoulder injury

Test	Valid N	Cohen’s Kappa (95% CI)	Agreement %
Inability to abduct > 90°^a^	42	0.90 (0.77–1.03)	95%
Painful arc	42	0.56 (0.36–0.76)	70%
Abduction strength	47	0.82 (0.64–0.99)	91%
Resisted abduction pain	48	0.50 (0.25–0.74)	75%
Hawkins` test	45	0.25 (0.01–0.48)	72%
External rotation reduced by ≥20°^a^	40	0.68 (0.42–0.93)	88%
External rotation strength	48	0.70 (0.48–0.92)	88%
Small finger test	48	0.51 (0.24–0.79)	81%
External rotation lag sign	39	0.40 (0.07–0.74)	89%
Belly-Press	45	0.63 (0.25–1.01)	93%
Internal rotation lag sign ¤	48	1 (1.00–1.00)	100%
Internal rotation lag sign anteriorly ^b^	46	–	96%
		**Linear weighted kappa (95% CI)**	
Internal rotation AROM (4 levels)	48	0.52 (0.29–0.74)	NA

^a^Derived from registered number of degrees of active range of motion; ¤ In 2 patients the first examiner registered “positive” and the second “not possible”; ^b^ The second assessors did not register any positive test

**Fig. 2:**
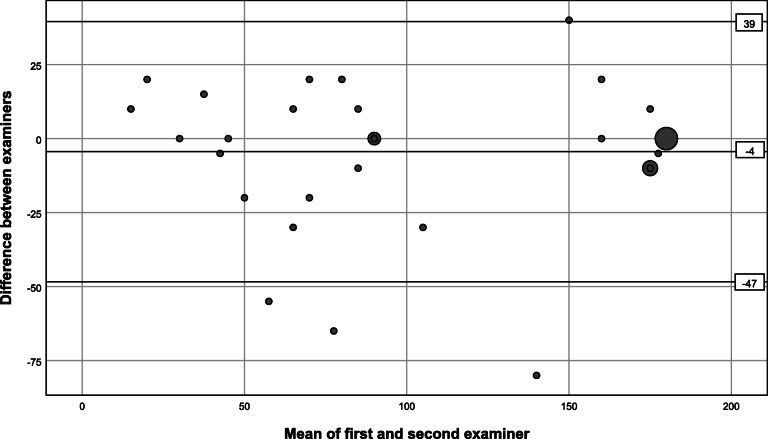
Bland Altman plot of degrees of active range of abduction. The horizontal lines represent the mean and ± 1.96 x SD of the difference between the first and second examiner. Six patients were not tested to maximum abduction due to recent glenohumeral dislocation. The largest marker represents 12 cases. The medium sized 4 and 3, respectively

**Fig. 3:**
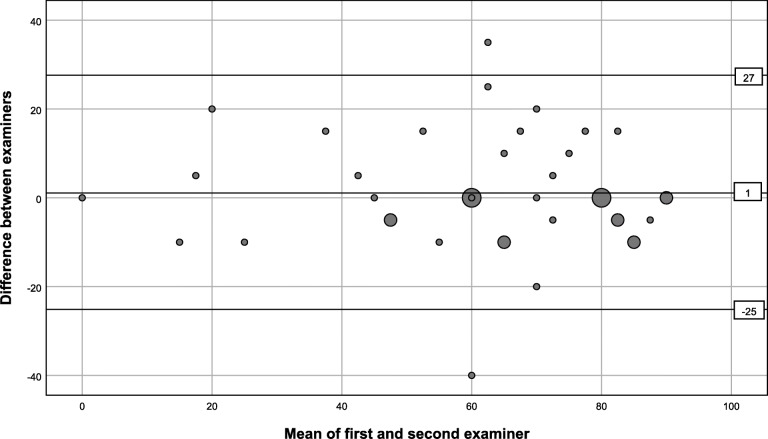
Bland Altman plot of degrees of active range of external rotation. The horizontal lines represent the mean and ± 1.96 x SD of the difference between the first and second examiner. Eight patients were not tested for maximum external rotation due to recent glenohumeral dislocation. The largest markers represent 3 cases, the medium sized 2

We observed excellent interrater agreement for the abduction strength test, substantial and moderate for external rotation strength assessed conventionally and by the small finger test, respectively. The internal rotation lag sign was categorized as positive in two patients by the first assessors, whereas the second assessors categorized it as not possible to perform (Table 2). There was full agreement between the first and second assessors regarding the remaining negative tests. No reliability values could be calculated for the internal rotation lag sign performed anteriorly to the body, as there was no positive finding registered by the second assessors. There was almost perfect agreement between the examiners for the inability to abduct > 90°, deduced from the registered number of degrees of abduction (Table 2), whereas agreement was substantial for registering a loss of external rotation ≥20° compared to the uninjured side. For abduction AROM the ICC (ICC (1,1); one-way random, single measures) confidence interval suggested good to excellent reliability, and for external rotation AROM moderate to excellent reliability (Table 3). Agreement of the continuous variables was further explored by quantifying the mean difference between the first and second assessor and the limits of agreement in Bland Altman plots (Figs. 2 and 3). The linear regression analyses did not indicate proportional bias. There was no obvious sign of heteroscedasticity in external rotation AROM (Fig. 3), but there could be a tendency for a narrower dispersion of values in abduction AROM at the high end of the spectre (Fig. 2).

**Table 3:** Reliability of continuous measurements

	Median (IQR)	ICC (95% CI)	SEM
Abduction AROM	100 (58–180)	0.93 (0.88–0.96)	15
External AROM	70 (45–80)	0.84 (0.72–0.91)	9

*AROM* active range of motion; *ICC* intraclass correlation coefficient (ICC (1,1); one-way random, single measures); *IQR* inter-quartile range; *SEM* standard error of measurement

## Discussion

The main result of the present study is that clinical assessment of active range of abduction and external rotation (expressed by the inability to abduct > 90° and external rotation reduced by ≥ 20° compared to uninjured side) and abduction and external rotation strength expressed best reliability among the included tests in patients in the acute phase of shoulder injury. According to Landis and Koch these results are classified as substantial to almost perfect [30]. There is however no universally agreed upon kappa value that indicates «acceptable» agreement, and careful interpretation is as always necessary. Others have used kappa > 0.60 or absolute agreement of 80% as indicative of acceptable agreement in clinical tests of the shoulder [32].

We observed great variation of interrater agreement between tests; with kappa values ranging from 0.25 to 0.90. The tests with the two lowest scores almost included zero in the confidence intervals (Table 2). The internal rotation lag sign had a kappa value of one, but there were only 6 patients with a subscapularis tear in the superior portion of the tendon and a strong predominance of negative tests. This result should therefore be interpreted with caution.

Absolute values of degrees of active range of abduction and external rotation were registered by the examiners. In addition, we dichotomized the values into the inability to abduct above 90° and reduction in external rotation ≥20° or more, as positive or negative tests. Interrater agreement was evaluated by ICCs and SEMs for assessment of the estimated number of degrees of active range of motion. The results presented in Table 3 indicate moderate to excellent reliability. In a previous study assessing the reliability of active range of motion in an identical way but performed by trained physical therapists, the ICC was 0.96 compared to 0.93 in our study, both excellent [32]. There could be a tendency for a narrower dispersion of the difference between the examiners in the Bland-Altman plot when abduction got close to normal (Fig. 2), indicating that heteroscedasticity may have been present. The kappa values were still excellent and good for the binary tests inability to abduct above 90° and external rotation reduced by ≥20°, respectively (Table 2). The finding of a high degree of agreement between the physicians when it comes to estimating active range of motion, is supported by a previous study on hip range of motion reporting high agreement between visual estimates and goniometer measurements with ICCs ranging from 0.80 to 0.88 [33].

The Hawkins` test for impingement may be a difficult test to perform and interpret in the acute setting where a considerable number of patients experience pain at elevation of the arm to shoulder level. This is illustrated by the lowest level of agreement of the present study, but still fair according to the Landis and Koch interpretation [30]. Cadogan and co-workers report similar fair values [24]. In Lange’s systematic review and meta-analysis extensive heterogeneity was observed for the Hawkins` test, and the results indicated an overall kappa value of 0.47 (moderate) [8].

There are several possible explanations to the variation in reliability among the tests in the present study. The most obvious is that for some tests more than others, the same signs and symptoms may be interpreted differently by different physicians. Second, the patient may experience a training effect resulting in a discrepancy between the findings of the first and second assessors. A patient having experienced pain may be more hesitant during the second testing, or unable to perform as well as the first time. Conversely, patients who perform the test without much pain may push their limit further the next time. Third, it is possible that providing more training of the physicians than what was offered in the present study could have improved reliability. The generalizability to emergency departments and primary health care would on the other hand have decreased, as the physicians would have been trained to be more similar to shoulder specialists than first line physicians.

In spite of the diversity, there was a tendency for tests estimating range of motion and strength to have superior reliability to tests interpreting pain (resisted abduction pain, Hawkins` test). This is in keeping with the results from a recent study reporting that for resisted external rotation; muscle weakness alone had better diagnostic validity for the detection of infraspinatus tears than pain or muscles weakness and/or pain [34].

Of the tests for which kappa values were calculated, 5 tests expressed substantial or excellent inter-rater reliability, whereas 5 expressed moderate reliability. The latter is not surprising in the light of the reliability reported in other clinical evaluations. A recent study examined interrater agreement for radiographic evaluation of glenohumeral osteoarthritis and found moderate kappa values of about 0.5 in experienced radiologists [35], whereas another two recent studies of shoulder examination techniques reported great diversity of the kappa values and wide confidence intervals [11, 29]. In the present study, several tests had wide confidence intervals, especially the belly-press test and the external rotation lag sign. The tests expressing the best kappa values also had narrower confidence intervals.

One of the strengths of the present study is that it provides data with external validity to facilities both in hospitals and primary care that admit the majority of acute shoulder injuries. The included patients were not referred, and the four physicians performing the tests were not shoulder specialists. Several authors have pointed out the lack of data on the performance of shoulder tests from such a setting, as most previous studies have involved referred patients examined by specialists [5, 12, 13].

The study has some limitations. First, intrarater reliability was not studied. Patients with acute shoulder injuries may experience changes in symptoms, making it necessary to keep the time interval between tests short. To adequately blind the physician to patients they examined hours earlier would have been challenging. Due to the methodological difficulties, only one of 18 studies in a recent review of the reliability of physical examination tests for shoulder pathologies reported intrarater reliability [8]. Second, as in other reliability studies examining shoulder tests, the confidence intervals were quite wide [7, 8, 11]. A higher number of included patients could possibly have reduced the confidence intervals. Finally, only six patients had full-thickness tears of the subscapularis, all limited to the superior portion of the tendon. The test results related to the subscapularis tendon should therefore be interpreted with caution.

## Conclusions

Kappa values were excellent for the inability to abduct > 90° and abduction strength and substantial for external rotation strength. There was a tendency for tests assessing pain to be less reliable than tests assessing range of motion and strength. Commonly used tests like the external rotation lag sign and Hawkins` test expressed the lowest kappa values of the included tests. Leaving these tests out from the examination in the acute phase of shoulder injury should be considered.

ICC for estimating active range of abduction and external rotation were acceptable and similar, but relative to the range, better for abduction than external rotation.

The present study contributes to filling the knowledge gap regarding the reliability of shoulder tests. As tests that do not measure consistently cannot be accurate, the results of the present study indicate which physical examination tests may be effective in detecting acute rotator cuff tears in patients during the acute phase of shoulder injury in the first line setting. Effective physical examination tests may improve the management of these patients both by providing a more reliable tool for the selection of patients for advanced imaging, as well as by providing the patient with a diagnosis and treatment plan at an earlier stage.

## Supplementary Information

**Additional file 1.**

**Additional file 2.**

## Data Availability

The dataset is available from the corresponding author upon reasonable request.

## Abbreviations

AROM: Active range of motion
ICC: Intraclass correlation coefficient
IQR: interquartile range
OSS: Oxford Shoulder Score
SD: Standard deviation
SEM: Standard error of measurement
