# Supplementary material for: Interrater reliability of physical examination tests in the acute phase of shoulder injuries
Source: BMC Musculoskelet Disord. 2021 Sep 9;22:770. doi: 10.1186/s12891-021-04659-x (PMC8427844; doi:10.1186/s12891-021-04659-x)
Supplement: Supplementary file 1 — Additional file 1. [file 12891_2021_4659_MOESM1_ESM.docx]

# Additional file 1

# FOLLOW-UP OF SOFT TISSUE INJURIES OF THE SHOULDER

(Excl dislocations)

### First visit

Patients with

- Negative conventional x-rays in two views

## and

- At least one of the following:
  - Active range of abduction reduced by > 30 degrees compared to the uninjured side by normal clinical examination, or significant loss of strength
  - Active range of external rotation reduced by > 20 degrees compared to the uninjured side by normal clinical examination, or significant loss of strength
  - Pain of 4 or more on a numeric rating scale

Should be offered follow-up 10 - 14 calendar days after the accident.

### Second visit (10 – 14 days)

Patients with

1. Pseudoparalysis (significantly reduced active range of motion or loss of strength regarding abduction (< 60 degrees), external rotation or internal rotation (belly-press) should be referred directly to MRI
2. progress, but who still fill the criteria for follow-up at the first visit, should be offered another follow-up no later than 4 weeks after the injury
3. no symptoms or return to pre injury pain and level of functioning do normally not need further follow-up

### Three or more consultations

Consider MRI in patients with ongoing pain or loss of function after acute injury
