# Supplementary material for: Interrater reliability of physical examination tests in the acute phase of shoulder injuries
Source: BMC Musculoskelet Disord. 2021 Sep 9;22:770. doi: 10.1186/s12891-021-04659-x (PMC8427844; doi:10.1186/s12891-021-04659-x)
Supplement: Supplementary file 2 — Additional file 2. [file 12891_2021_4659_MOESM2_ESM.doc]

# Additional file 2

# Instructions to physicians participating as examiners in the study

The patient should preferably be standing up when examined. If difficult for the patient, he or she may be examined sitting down.

In patients with a verified or suspected glenohumeral dislocation, abduction of more than 90° or tests implying maximal external rotation should not be executed.

|  | TEST | DESCRIPTION | INTERPRETATION | REGISTRATION | TIPS |
| --- | --- | --- | --- | --- | --- |
| **1** | ABDUCTION AROM | Ask the patient to lift both arms to the side and towards the roof (demonstrate in the scapula plane, i.e. 20 - 30 degrees forwards from the coronal plane) with thumbs up, then down again. Stop at 90° if GH dislocation <6 weeks | Pain is not registered, apart from painful arc, see below. | Number of degrees | Start by considering 30-45-60-90-120 that are easily estimated, and adjust from there |
| **2** | PAINFUL ARC | As above | Pain during the arc from 60-120° is a positive test. Should be distinctly less painful in the beginning of the movement and from about 120 and up. The pain should be localised laterally on the upper arm in the deltoid region, if positive ask where it hurts.  Negative: Abduction all the way up without a painful arc from 60-120, but may report pain, for example all the way up or only during last part of motion. | Pos  Neg  Not possible |  |
| **3** | ABDUCTION STRENGTH | Upper arm along side, 90° elbow flexion, test both at 0° and 45° abduction. Put your hand not providing resistance on the patient`s contralateral shoulder for support, and ask the patient to raise the arm against resistance. | Normal strength injured shoulder = strength indistinguishable to uninjured side | Normal  Reduced | Do not consider possible causes (whether due to pain etc),  just whether strength is reduced or not |
| **4** | RESISTED ABDUCTION  PAIN | As above, but position the arm at about 20-30° abduction. Support the patient`s contralateral shoulder. Is there pain in this position just by keeping the arm in the position? At isometric resistance? At eccentric resistance? |  | Pain against  - gravity alone  -isometric force  -eccentric force  No pain |  |
| **5** | HAWKINS IMPINGEMENT SIGN | Position the shoulder and elbow in 90° flexion. Stabilise the scapula by supporting the scapular spine with fingers 2-5 and the thumb over the lateral margin. Stabilise elbow with the other hand and rotate the upper arm internally | Pain or distinct worsening of pain during internal rotation when the greater tubercle is rotated under the coracoacromeal arc is a positive test. | Pos  Neg  Not possible | If the patient experiences distinct pain by moving the arm up to 90° flexion, the test is also positive |
| **6** | EXTERNAL ROTATION AROM | Patient stands with back against wall. Elbows flexed to 90° and along side of body. Stand in front of the patient and demonstrate. Do not perform if GH disl <6 weeks | Pain is not registered | Number of degrees | 0 – lower arm straight forwards  90° – parallel to the wall |
| **7** | EXTERNAL ROTATION STRENGTH CONVENTIONAL | The patient`s shoulder should be in the neutral position (90° in elbows and lower arm in the sagittal plane, i.e. straight forwards). Stand facing patient and provide resistance to the patient`s wrists with your hands | Pain is not registered | Normal  Reduced | Do not consider possible causes (whether due to pain etc),  just whether strength is reduced or not |
| **8** | EXTERNAL ROTATION STRENGTH SMALL FINGER TEST | Elbows flexed to 90°and along body, thumbs up. Stand on patient`s side and ask him or her to resist the force when you push against the patient`s wrist using your small finger, attempting to internally rotate the shoulder | Pain is not registered | Normal  Reduced |  |
| **9** | EXTERNAL ROTATION  LAG SIGN | Stand with back against wall. Elbows flexed at 90°. Support with one hand under elbow and elevate (scapula plane) the arm slightly (20°). The other hand externally rotates the arm by the wrist to maximum position, let up slightly, about 5°. Ask the patient to keep the position when let go of the wrist, but still support the elbow | Positive if unable to hold position | Pos  Neg  Not possible | Pain is not noted, but if pain hinders execution of the test, note as not possible |
| **10** | INTERNAL ROTATION AROM  (HAND ON BACK) | The patient tries to move the hand to the back, if possible the thumb as high along the spine as possible. Healthy side first. | Pain is not registered | 0-plane – back pocket – lumbar area – inter-scapular area |  |
| **11** | BELLY-PRESS  (INTERNAL ROTATION STRENGTH) | Patient presses flat hand against belly attempting to keep arm in a maximally internally rotated position. The physician may then provide resistance by pulling the arm off the belly (external rotation) | Positive test if (1) elbow glides behind body and the patient presses against belly by extending the shoulder and flexing the wrist, or (2) reduced strength compared to healthy side  Negative/normal strength: Elbow does not glide back and wrist is not flexed, i.e. the elbow is in front of the body with straight wrist and indistinguishable strength compared to healthy side | Pos  Neg  Not possible |  |
| **12** | INTERNAL ROTATION LAG SIGN BEHIND BACK(LIFT-OFF TEST) | The examiner extends and internally rotates the arm to maximum position with elbow flexed, i.e. hand off the lumbar spine. Ask the patient to hold the position and let go | Positive if unable to hold position | Pos  Neg  Not possible | Pain is not noted, but if pain hinders execution of the test, note as not possible |
| **13** | INTERNAL ROTATION LAG SIGN IN FRONT | Ask the patient to put both hands on the belly, while you lead both elbows passively forwards to the maximally tolerable internal rotation of the shoulder. Ask the patient to hold the position and let go | Positive if unable to hold position | Pos  Neg  Not possible |  |
